# The impact of emotional intelligence and personality traits on the occurrence of unsafe behaviors and needle stick injuries among the nurses

**DOI:** 10.1016/j.heliyon.2022.e09584

**Published:** 2022-05-30

**Authors:** Masoud Askari Majdabadi, Saeid Yazdanirad, Rasoul Yarahmadi, Jamileh Abolghasemi, Hossein Ebrahimi

**Affiliations:** a Department of Occupational Health Engineering, School of Public Health, Iran University of Medical Sciences, Tehran, Iran; b School of Health, Shahrekord University of Medical Sciences, Shahrekord, Iran; c Department of Epidemiology and Biostatistics, School of Public Health, Iran University of Medical Sciences, Tehran, Iran

**Keywords:** Needlestick injuries, Emotional intelligence, Personality traits, Safe behavior

## Abstract

This study was aimed to investigate the effect of emotional intelligence and some personality traits on safe behavior and needle stick injuries among the nurses. This cross-sectional study was performed on 200 nursing staff of a hospital in Iran. To collect data, individuals were asked to complete several questionnaires, including demographic questionnaire, domain-specific risk-taking questionnaire, Rosenberg self-confidence questionnaire, Buss-Perry aggression questionnaire, Goleman emotional intelligence questionnaire, and safe behavior questionnaire. Also, the number of needlestick injuries in the participants was extracted from their medical records. Data were analyzed using the SPSS software (version 22), and path analysis was performed using AMOS software. The prevalence of needle stick injuries in the subjects was estimated by 45.5 percent. The results showed that increasing risk-taking, increasing aggression, decreasing self-confidence, and decreasing emotional intelligence reduced safe behavior and increased the number of needle injuries (P < 0.001). In the present study, some personal traits affecting the occurrence of needlestick injuries were identified. It is recommended that people without these negative traits are applied in dangerous occupations with a high probability of needle stick injuries.

## Highlights

• The prevalence of needle stick injuries in the nurses was high.
• Increasing risk-taking increased the number of needle injuries.
• Increasing aggression increased the number of needle injuries.
• Decreasing self-confidence increased the number of needle injuries.
• Decreasing emotional intelligence increased the number of needle injuries.

## 1 Introduction

There are various agents to threaten human health in the world (Rafiemanesh et al., 2016). Some of the harmful factors are related to work environments (Gharibi et al., 2021; Kalteh et al., 2018). The staff of health centers exposes to several occupational hazards, such as needle sticks (Bouya et al., 2020). Needlestick is one of the biggest occupational hazards to threaten the health of personnel in hospitals. If the needle is contaminated with the patient's blood and secretions, it is very dangerous and can lead to the transmission of some diseases, such as hepatitis B, hepatitis C, AIDS, brucellosis, gonorrhea, Herpes, malaria, and syphilis (Ifeanyi et al., 2018). Based on the reports of the World Health Organization (WHO), 40% of hepatitis B and C infections and 2.5% of HIV infections occur because of needle stick injuries in health care workers (Organization, 2002). Statistics indicate that each of the health care workers averagely experiences four needle sticks at one year (Yarahmadi et al., 2014). In the United States, four hundred thousand persons among four million health care workers suffer from needle stick injuries every year (Mirzaee and Ravari, 2003). In developing countries of the middle east, the condition is worse (Salehiniya et al., 2016). the results of a study show that the statistic of needle stick injuries in these countries is nearly 50 percent (Zafar et al., 2008). Based on the results of a study performed by Rezaei et al., the one-year prevalence of needle stick injuries among Iranian nurses was 44 percent (Rezaei et al., 2017). The prevalence of needle sticks is different, and it occurs more frequently among nurses, surgeons, and emergency personnel (Alhazmi et al., 2018; Reddy et al., 2017). Of them, the greatest risk is related to nurses, who possess up to 50% of all needlestick injuries (Jahangiri et al., 2016; Ozdelikara and Tan, 2012). To prevent these injuries, the main causes must first be identified (Mokarami et al., 2020; Raeisi Shahraki et al., 2016). Previous studies have investigated some of these causes. The human factor is one of the agents affecting the occurrence of injuries (Khoshakhlagh et al., 2017). According to Heinrich's theory, 80 percent of injuries happen because of unsafe behavior (Khoshakhlagh et al., 2021). For this reason, developed countries have focused on controlling this factor in recent years (Khoshakhlagh et al., 2019). This altered approach has created the science of behavior-based safety (Mohammadfam et al., 2017). This type of behavior is defined as actual actions performed by workers based on the safety guidelines (Pourabdian et al., 2020). All personal behaviors related to the organization's safety are considered as safety performance (Mahdinia et al., 2017). Emotional intelligence, as a subset of social intelligence, is one of the parameters to determine the behavior type of an individual under the existing situation (Kotsou et al., 2019). It describes the ability to control the feelings and emotions of oneself and others and use this information for the guidance of thoughts and actions (Raghubir, 2018). It determines an individual's respect toward the rights of other people (Prentice et al., 2020). The personality, as an internal factor in humans, is another important parameter. It includes unique features formed during the time, which distinguishes a person from another (Alessandri et al., 2020). Personality traits can affect high-risk behaviors and people's perceptions of health and danger (Pourmazaherian et al., 2021). Some researchers believe that extroverts possess a high degree of sociability and perform more risky behaviors for achieving the desired level of arousal (Alessandri et al., 2020). Given that arousal and self-evident and bold behaviors are part of the characteristics of. So excitement, self-representation, and boldness are part of the characteristics of extroverts (Pourmazaherian et al., 2021). Based on the results of the studies, excitement, risk-taking, adventure, and high arousal have a significant relationship with the violations (Bucsuházy et al., 2020). Moreover, risky behaviors are associated with traits such as anxiety, aggression, anger, negative emotions, emotional instability, and other neurotic personality traits (Tao et al., 2017). Although several studies have examined the effect of personality traits on the occurrence of injuries, the findings are contradictory. Also, the previous studies have not evaluated the role of emotional intelligence and personality traits on the occurrence of needlestick injuries. Therefore, the present study was aimed to investigate the impact of these factors on the occurrence of unsafe behaviors and needle stick injuries among the nurses.

## 2 Materials and methods

### 2.1 Participants

This cross-sectional study was performed on 200 nurses in one of the Iranian hospitals. Inclusion criteria included having at least two years of career length, working in the nursing ward, and not having serious physical and mental health problems. Exclusion criteria were the non-cooperation of the participants. The 345 nurses were working in this hospital, and the 273 subjects had inclusion criteria. Based on the Cochran formula, the required sample size was calculated by 182 persons. However, given that the minimum samples required in structural equation modeling (SEM) is 200 subjects, 200 nurses participated in this study (Westland, 2010). The subjects were randomly selected from different sections.

### 2.2 Data collection

Before beginning the study, the aims and steps of this study were explained to the subjects. This study was conducted based on the established ethical guidelines, and participants signed the consent form. Ethical approval for performing this study was obtained from the institutional research ethics committee of Iran University of Medical Sciences (IR.IUMS.REC.1398.761). Moreover, the study complies with all regulations in the methods section of the paper. All participants were asked to complete several questionnaires during rest times. Moreover, the number of needlestick injuries of the participants in the last year was extracted from their medical records. Persons with needle stick injuries are defined as people who have experienced the accidental puncture of the skin with a syringe in the last year (Shah et al., 2006).

### 2.3 Tools

The required information was gathered by several tools, including demographic questionnaire, domain-specific risk-taking questionnaire, Rosenberg self-confidence questionnaire, Buss-Perry aggression questionnaire, Goleman emotional intelligence questionnaire, and safe behavior questionnaire. These questionnaires were selected to gather data because those are well-known tools with acceptable validity and reliability and are frequently used around the world. Also, those have been translated into the Persian language, and their validity and reliability have been evaluated in Iran.

#### 2.3.1 Demographic questionnaire

This questionnaire included general data such as age, work experience, shift number per month, and ward. This questionnaire is available in Annex A.

#### 2.3.2 Domain-specific risk-taking questionnaire

This questionnaire has 30 items with a seven-point Likert scale from completely unlikely (score 1) to quite probable (score 7). In the study of Moradi et al., the validity of the questionnaire was confirmed. Also, Cronbach's alpha coefficient of this tool was obtained by 0.7 (Moradi et al., 2014). This questionnaire is available in Annex B.

#### 2.3.3 Rosenberg self-confidence questionnaire

This questionnaire has 10 items with a four-point Likert scale. Alizadeh et al. confirmed the validity and reliability of this questionnaire. They reported that Cronbach's alpha coefficient of this tool was equal to 0.83 (Alizadeh et al., 2005). This questionnaire is available in Annex C.

#### 2.3.4 Buss-Perry aggression questionnaire

This questionnaire consists of 29 items with a five-point Likert scale. The results of a study performed by Samani showed that this tool had acceptable validity and reliability. Using the test-retest method, the reliability coefficient was reported by 0.78 (Samani, 2008). This questionnaire is available in Annex D.

#### 2.3.5 Goleman emotional intelligence questionnaire

The emotional intelligence test of Siberia Schering was used. It has comprised of two parts with 70 questions. The first and second parts have been designed based on the various life situations and emotional stories, respectively. The subjects answer the questions by five-point Likert. Saeida et al. evaluated the validity and reliability of the version translated of the questionnaire. The seven questions of the first part were omitted due to the low correlations with the total score, and the second part was completely deleted because of the inconsistency of the stories with Iranian culture. They concluded that the internal consistency of the questionnaire with 33 items was equal to 0.85 (Saeida-Ardakani et al., 2012). This questionnaire is available in Annex E.

#### 2.3.6 Safe behavior questionnaire

Mahdinia et al. have designed and validated this questionnaire to evaluate safety behavior in Iran. They concluded that the questionnaire designed with 12 questions on safety implementation and 11 questions on safety participation had good reliability (Mahdinia et al., 2016). This questionnaire is available in annex F.

### 2.4 Data analysis

Firstly, data were entered into the software of statistical package for the social sciences (SPSS) (version 22). Then, the skew and kurtosis curves were used to evaluate the normality of the studied parameters. Given the normal distribution in all variables, the correlation between the variables was examined using the Pearson test. Also, the multicollinearity was checked among the independent variables. After that, structural equation modeling (SEM) was used to perform path analysis. For this purpose, a theoretical model was drawn in AMOS software and the relations between the variables were investigated. The path analysis was used to study the direct and indirect effects of the variables on the number of needlestick injuries. Given the aim of the study, in the drawn model, risk-taking, self-confidence, aggression, and emotional intelligence were considered as independent variables. The number of needle stick injuries and safe behavior also were dependent and moderator variables, respectively. based on the assumptions, all possible paths were depicted between the variables, and the paths related to the insignificant relations were omitted. The fitness of the model was also evaluated using absolute, comparative, and normed fitness indicators.

## 3 Results

This descriptive-analytical study was performed on 200 nurses in one of the hospitals of Iran University of Medical Sciences. The participants were working in the various wards, including surgery (16 subjects), ICU (25 subjects), pediatrics (28 subjects), internal medicine (17 subjects), operating room (18 subjects), oncology (21 subjects), emergency (30 subjects), CCU (12 subjects), psychiatry (21 subjects), and nephrology (12 subjects). Of them, 23 persons were nursing supervisors and 177 individuals were ward nurses. Among them, 108 subjects (53.5%) did not experience needlestick injuries and 92 persons (45.5%) possessed a history of the injuries during work shifts. The values of mean ± standard deviation of the age, work experience, and shift number per month in the participants were 34.00 ± 6.70, 9.50 ± 5.99, and 25.70 ± 4.13, respectively. Moreover, Table 1 represents the statistical distribution of demographic characteristics in two groups with and without needlestick injuries. The results of the independent t-test showed that mean values of age and work experience in subjects with needle stick injuries compared to individuals without needle stick injuries were significantly lower (P < 0.037). The values of mean ± standard deviation of the risk-taking, self-confidence, aggression, emotional intelligence, safe behavior, and the number of needlestick injuries were 112.44 ± 24.07, 11.67 ± 4.40, 49.63 ± 14.46, 103.08 ± 9.83, 86.32 ± 15.33, and 1.59 ± 1.42, respectively. Furthermore, Table 2 reports the statistical distribution of the studied variables in two groups with and without the needle stick experience. The results of the independent t-test indicated that mean values of risk-taking, self-confidence, aggression, and safe behavior were significantly different between the two groups with and without needle stick injuries (P < 0.001).

**Table 1:** The statistical distribution of demographic characteristics of the subjects with and without needle stick experience.

Variable	Subjects without needle stick injuries (n = 108)			Subjects with needle stick injuries (n = 92)			P value
	Range	Mean	Standard deviation	Range	Mean	Standard deviation	
Age	24–53	35.44	7.62	24–46	32.30	5.62	0.026
Work experience	2–29	10.61	6.78	1–21	8.20	5.06	0.037
Shift number per month	17–32	25.62	4.18	17–32	25.79	4.08	0.235

**Table 2:** The statistical distribution of the studied variables.

Variable	Subjects without needle stick injuries (n = 108)			Subjects with needle stick injuries (n = 92)			P value
	Range	Mean	Standard deviation	Range	Mean	Standard deviation	
Risk taking	37–189	105.16	29.14	71–163	121.00	18.11	<0.001
Self-confidence	2–15	8.67	3.83	6–26	15.19	5.06	<0.001
Aggression	13–76	45.20	12.81	18–86	54.84	16.39	<0.001
Emotional intelligence	86–138	104.19	11.88	86–116	101.78	7.42	0.093
Safe behavior	32–110	90.45	15.86	37–105	81.47	14.70	<0.001

Table 3 reports the correlation coefficients between the quantitative variables. Based on the results, there were significant positive relationships between the number of needle stick injuries with the risk-taking (r = 0.239) and aggression (r = 0.415) in the subjects under study (P < 0.01). Also, the meaningful inverse relationships were observed between the number of needle stick injuries with self-confidence (r = - 0.731) and safe behavior (r = - 0.364) (P < 0.01). Moreover, safe behavior had a positive correlation with emotional intelligence (r = 0.483) and the negative correlations with the variables of number of needle stick injuries (r = - 0.364), risk-taking (r = - 0.425), and aggression (r = - 0.226) (P < 0.01).

**Table 3:** Correlation coefficients between the quantitative variables.

Variable		1	2	3	4	5	6
1	Number of needle stick injuries	-					
2	Risk-taking	0.239∗∗	-				
3	Self-confidence	- 0.731∗∗	- 0.191∗∗	-			
4	Aggression	0.415∗∗	0.270∗∗	- 0.347∗∗	-		
5	Safe behavior	- 0.364∗∗	- 0.425∗∗	0.026	- 0.226∗∗	-	
6	Emotional intelligence	- 0.050	- 0.158∗	0.076	0.035	0.483∗∗	-

∗∗P < 0.01.

∗P < 0.05.

**Figure 1:**
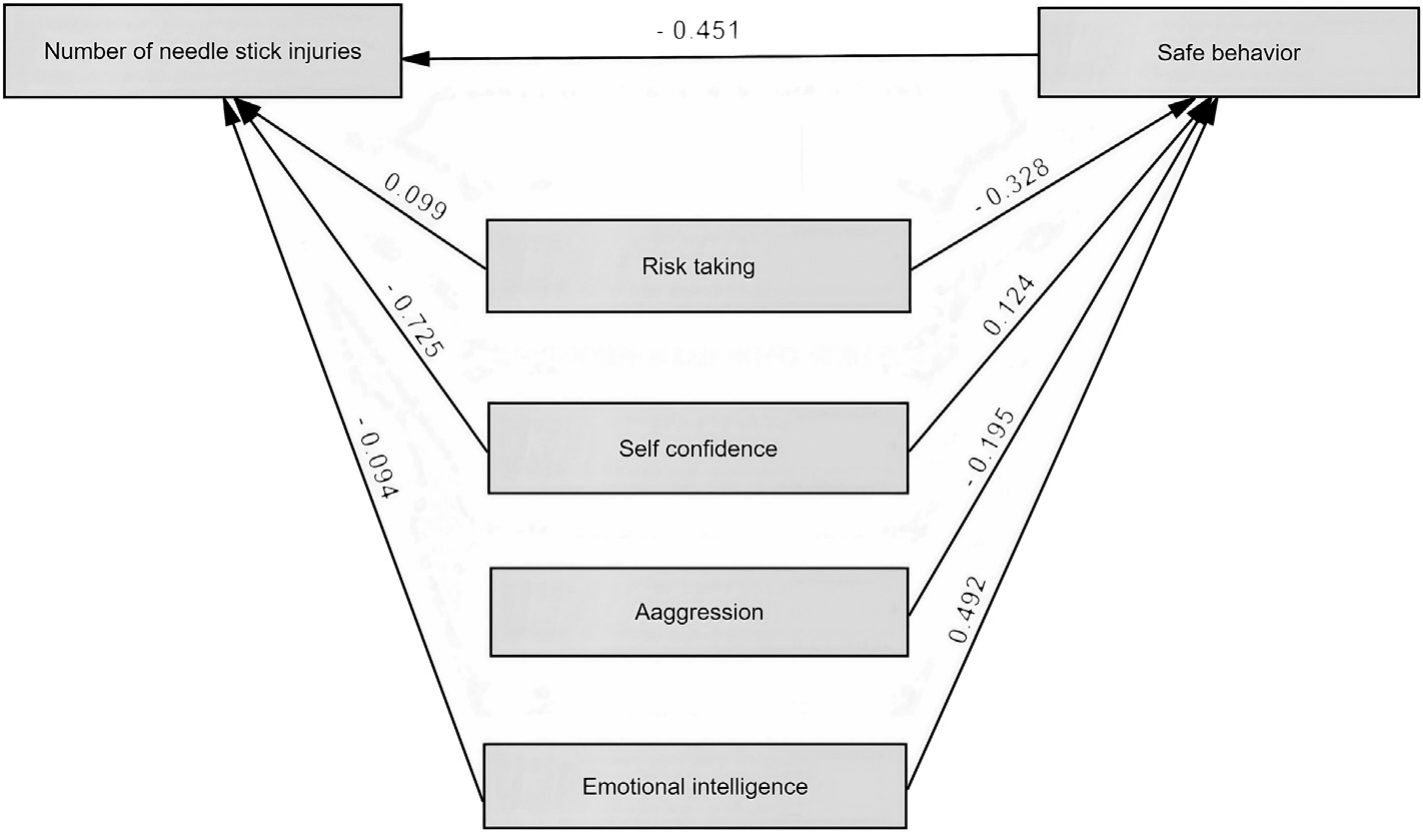
The theoretical model analyzed by structural equation modeling (SEM).

Figure 1 indicates the theoretical model analyzed by structural equation modeling (SEM). Also, Table 4 represents the effect coefficients of the studied variable on the number of needlestick injuries. Based on the results, the variables of risk-taking, self-confidence, aggression, emotional intelligence, and safe behavior had a direct effect on the number of needlestick injuries. The highest and lowest direct effects were related to the variables of self-confidence (- 0.725) and emotional intelligence (- 0.094), respectively. However, the results showed that the highest and lowest indirect effects were assigned to the variables of emotional intelligence (- 0.221) and self-confidence (- 0.055), respectively. Moreover, the variables of self-confidence (- 0.780) and aggression (0.087) indicated the highest and lowest total effects on the number of needlestick injuries. Table 5 also reports the goodness-of-fit indices of the theoretical model. The results revealed that all obtained values of goodness-of-fit indices are in optimal ranges.

**Table 4:** The effect coefficients of the studied variable on the number of needle stick injuries.

Variable	Direct effect	Indirect effect	Total effect
Risk taking	0.099	0.147	0.246
Self-confidence	- 0.725	- 0.055	- 0.780
Aggression	-	0.087	0.087
Emotional intelligence	- 0.094	- 0.221	- 0.315
Safe behavior	- 0.451	-	- 0.451

**Table 5:** The goodness-of-fit indices of the theoretical model.

Index	Name	Threshold of Fitness	Obtained value
Absolute fitness indices	Goodness-of-fit index (GFI)	>0.9	0.928
	Adjusted goodness-of-fit index (AGFI)	>0.9	0.909
Comparative fitness indices	Normed fit index (NFI)	>0.9	0.917
	Comparative fit index (CFI)	>0.9	0.935
	Incremental fit index (IFI)	0–1	0.931
Normed fit index	Root mean squared error of approximation (RMSEA)	<0.1	0.069
	Normed Chi-square (X^2^/df)	1–3	2.785

## 4 Discussion

prevalence of needle stick injuries among the participants was equal to 45.5%. In the study of Ghasemi et al., this value among nurses of an Iranian hospital was estimated by 41.2% (Ghasemi et al., 2017). Also, Rezaei et al. concluded that the one-year periodic prevalence of needle sticks among Iranian nurses was 44% (Rezaei et al., 2017). Moreover, the results of a study performed by Yarahmadi et al. showed that 40.42% of health care workers in an Iranian hospital suffer from needle stick injuries (Yarahmadi et al., 2014). The results of the present study are consistent with those of other studies, which indicate the high prevalence of needle sticks in Iran. Given its importance and dangerous consequences, Effective factors must be identified to prevent the occurrence of these injuries.

The results of the present study showed that the mean values of the studied variables, including self-confidence, aggression, safe behavior, risk-taking, and emotional intelligence, were significantly different between the two groups with and without needle stick injuries. So the results revealed that increasing risk-taking, increasing aggression, decreasing self-confidence, and decreasing emotional intelligence reduced safe behavior. Also, based on the results, the variables of risk-taking, self-confidence, and emotional intelligence could, directly and indirectly, affect the number of needle stick injuries through decreasing safe behavior. The aggression had only an indirect effect on the number of needlestick injuries. Increasing safe behavior resulted in decreasing number of needle stick injuries. Among variables, the highest and lowest direct effects were related to the variables of self-confidence and emotional intelligence, respectively. Also, the highest and lowest indirect effects were assigned to the variables of emotional intelligence and self-confidence, respectively. Moreover, the variables of self-confidence and aggression showed the highest and lowest total effects on the number of needlestick injuries. People with risk-taking traits pay less attention to the consequences of their behavior, and it causes them to perform dangerous actions. Identifying these persons in the workplace and controlling their behavior can reduce injuries. The results of a study carried out by Dahlgren et al. showed that the subjects with risk-taking traits commit more dangerous behaviors. These actions can make irreparable consequences for them (Dahlgren et al., 2009). Mcnabb and Keller also concluded that nurses with higher risky behaviors are exposed to the hazard of HIV transmission in their workplace (McNabb and Keller, 1991). Moreover, Man et al. demonstrated that risk-taking traits among construction workers could lead to adverse consequences and severe injuries (Man et al., 2017). Therefore, people with the risk-taking trait in workplaces and hospitals have more proneness for performing risky behaviors. Aggression, as another important trait, impress on the type of behavior. Mood and aggression influence function, mental process, risk perception, and data restoration in humans. The results of some studies indicate that people with a positive mood compared to others possess more friendly, cooperative, supportive, and safe behavior. A good mood also increases job performance and satisfaction. If people have a negative mood, they are more likely to remember negative events and situations. People with a negative mood use lower information for decision-making, paying attention, and finding the right solution. They overestimate the risk of the negative events and underestimate their ability to decrease this risk (Miner and Glomb, 2010). Jiang et al. concluded that verbal and physical aggression can significantly predict the injuries at workplace (Jiang et al., 2018). Sani et al. also resulted that the variables of aggression and emotional self-regulation can forecast risky behaviors (Sani et al., 2017). The results of the present study showed that aggression could indirectly increase the number of needle stick injuries through increasing unsafe behaviors. This may be because aggressive persons have less focus on their behavior and are more likely to commit errors. Also, they may perform unsafe actions due to antisocial behavior. Moreover, low self-confidence was identified as another effective trait in the present study. This trait reflects the individual ability to perform the desired behavior. If health care staffs are unable to follow safety instructions, the increased likelihood of needle stick injuries is reasonable. In the present study, this trait could, directly and indirectly, increase the number of needlestick injuries. Jitwasinkul et al. represented a Bayesian model to improve the safe behaviors and concluded that high self-confidence among construction workers can increase their safe actions (Jitwasinkul et al., 2016). The results of a study performed by Ghimire et al. also showed that the prevalence of needle stick injuries was strongly associated with depression and social issues among medical personnel (Ghimire et al., 2017). Furthermore, the results of the present study revealed that increasing emotional intelligence could, directly and indirectly, reduce the number of needlestick injuries. Previous studies show that people with high emotional intelligence can control their excitements, distinguish the positive and negative consequences, and use their emotional information (Mayer et al., 2004). Therefore, persons with high emotional intelligence can correctly think, decide, and behave in insensitive and dangerous situations. Jeffries et al. concluded that this trait can positively impress on safety attitude and behavior among people (Jeffries, 2011). Also, Fallahi et al. demonstrated that drivers with high emotional intelligence exhibit safer behaviors (Falahi and Goudarzi, 2015). The results of the present study are consistent with those of previous studies.

As one of the limitations of the present study, these relations were not investigated among other health care workers. Therefore, it was suggested that other health care personnel are studied in the next research. Another limitation of this study was the lack of investigating the effect of demographic items in the model. Hence, it was proposed that the impact of these items, as a separate factor, is investigated in the next studies.

## 5 Conclusion

The results of this study showed that the prevalence of needle stick injuries among the nurses of the studied hospital was high. Based on the results, increasing risk-taking, increasing aggression, decreasing self-confidence, and decreasing emotional intelligence can increase unsafe behaviors and the number of needlestick injuries. Therefore, it is recommended that people without these negative traits are applied in dangerous occupations with a high probability of needle stick injuries.

## Declarations

### Author contribution statement

Masoud Askari Majdabadi & Hossein Ebrahim: Conceived and designed the experiments; Performed the experiments.

Saeid Yazdanirad: Conceived and designed the experiments; Wrote the paper.

Rasoul Yarahmadi: Contributed reagents, materials, analysis tools or data.

Jamileh Abolghasemi: Analyzed and interpreted the data.

### Funding statement

The authors did not receive any funding.

### Data availability statement

The authors do not have permission to share data.

### Declaration of interests statement

The authors declare no conflict of interest.

### Acknowledgement

Researchers need to thank all participants in this study.

### Additional information

No additional information is available for this paper.

## Appendix A Supplementary data

This study was conducted according to established ethical guidelines, and informed consent obtained from the participants. Ethical approval for this study was obtained from the institutional research ethics committee of Iran University of Medical Sciences (IR.IUMS.REC.1398.761).

safety behavior 2 safety behavior 2

rosenberg self confidence questionnire rosenberg self confidence questionnire

risk taking questionnaire risk taking questionnaire

Goleman_emotional_intelligence_test_-_2-10-12 Goleman_emotional_intelligence_test_-_2-10-12

demographic questionnire demographic questionnire

BussPerry_agression_questionnaire_scoring BussPerry_agression_questionnaire_scoring
