# Supplementary material for: The impact of emotional intelligence and personality traits on the occurrence of unsafe behaviors and needle stick injuries among the nurses
Source: Heliyon. 2022 May 30;8(6):e09584. doi: 10.1016/j.heliyon.2022.e09584 (PMC9344315; doi:10.1016/j.heliyon.2022.e09584)
Supplement: safety behavior 2 [file mmc1.pdf]

	Question	Response				
1	I follow the instructions and safety rules related to my work.	Never	Rarely	Sometimes	Often	Always
2	Under the influence of co-workers or work environment conditions, I violate safety rules and regulations.	Never	Rarely	Sometimes	Often	Always
3	Under the influence of management pressure, I violate safety rules and instructions.	Never	Rarely	Sometimes	Often	Always
4	I ignore safety rules and instructions due to work pressure or due to lack of time.	Never	Rarely	Sometimes	Often	Always
5	By ignoring some rules, I do some things better and faster.	Never	Rarely	Sometimes	Often	Always
6	I do my job without haste and at a safe speed.	Never	Rarely	Sometimes	Often	Always
7	I take safety warnings and signs seriously.	Never	Rarely	Sometimes	Often	Always
8	I use all personal protective equipment and safety equipment to do my job.	Never	Rarely	Sometimes	Often	Always
9	In doing my job, I use appropriate and healthy tools and equipment.	Never	Rarely	Sometimes	Often	Always
10	Because of my mastery and high experience in my work, I ignore safety tips.	Never	Rarely	Sometimes	Often	Always
11	I take responsibility for doing dangerous things and I do.	Never	Rarely	Sometimes	Often	Always
12	I keep my work environment clean and tidy.	Never	Rarely	Sometimes	Often	Always
13	I encourage my co-workers to work safely.	Never	Rarely	Sometimes	Often	Always
14	I work hard to improve the safety of my work environment.	Never	Rarely	Sometimes	Often	Always
15	I actively participate in safety sessions.	Never	Rarely	Sometimes	Often	Always
16	I report significant safety issues to management.	Never	Rarely	Sometimes	Often	Always
17	I help my co-workers when they are working in dangerous situations.	Never	Rarely	Sometimes	Often	Always
18	I act in such a way that safety programs are promoted and promoted within the company.	Never	Rarely	Sometimes	Often	Always
19	I voluntarily perform tasks or activities that help improve safety in my work environment.	Never	Rarely	Sometimes	Often	Always
20	In safety meetings, I present and state the points I want.	Never	Rarely	Sometimes	Often	Always
21	I report dangerous situations to the supervisor or safety representative.	Never	Rarely	Sometimes	Often	Always
22	To perform a specific task, I request information about the risks from the supervisor, safety representative or co-workers.	Never	Rarely	Sometimes	Often	Always
23	To improve the safety situation, I suggest the corrective measures I want to the supervisor or safety officer.	Never	Rarely	Sometimes	Often	Always
