# Supplementary material for: The impact of emotional intelligence and personality traits on the occurrence of unsafe behaviors and needle stick injuries among the nurses
Source: Heliyon. 2022 May 30;8(6):e09584. doi: 10.1016/j.heliyon.2022.e09584 (PMC9344315; doi:10.1016/j.heliyon.2022.e09584)
Supplement: rosenberg self confidence questionnire [file mmc2.pdf]

Annex C

Rosenberg self-esteem scale

		Strongly Disagree	Disagree	Agree	Strongly Agree
1.	On the whole, I am satisfied with myself.	0	1	2	3
2.	* At times, I think I am no good at all.	0	1	2	3
3.	I feel that I have a number of good qualities.	0	1	2	3
4.	I am able to do things as well as most other people.	0	1	2	3
5.	* I feel I do not have much to be proud of.	0	1	2	3
6.	* I certainly feel useless at times.	0	1	2	3
7.	I feel that I'm a person of worth, at least on an equal plane with others.	0	1	2	3
8.	* I wish I could have more respect for myself.	0	1	2	3
9.	* All in all, I am inclined to feel that I am a failure.	0	1	2	3
10.	I take a positive attitude toward myself.	0	1	2	3

* Items 2, 5, 6, 8, and 9 have reverse scores.
