# Supplementary material for: The impact of emotional intelligence and personality traits on the occurrence of unsafe behaviors and needle stick injuries among the nurses
Source: Heliyon. 2022 May 30;8(6):e09584. doi: 10.1016/j.heliyon.2022.e09584 (PMC9344315; doi:10.1016/j.heliyon.2022.e09584)
Supplement: risk taking questionnaire [file mmc3.pdf]

Annex B

Domain-Specific Risk-Taking (Adult) Scale – Risk Taking

For each of the following statements, please indicate the **likelihood** that you would engage in the described activity or behavior if you were to find yourself in that situation. Provide a rating from *Extremely Unlikely* to *Extremely Likely*, using the following scale:

1	2	3	4	5	6	7
Extremely Unlikely	Moderately Unlikely	Somewhat Unlikely	Not Sure	Somewhat Likely	Moderately Likely	Extremely Likely

1. Admitting that your tastes are different from those of a friend. (S)
2. Going camping in the wilderness. (R)
3. Betting a day's income at the horse races. (F/G)
4. Investing 10% of your annual income in a moderate growth diversified fund. (F/I)
5. Drinking heavily at a social function. (H/S)
6. Taking some questionable deductions on your income tax return. (E)
7. Disagreeing with an authority figure on a major issue. (S)
8. Betting a day's income at a high-stake poker game. (F/G)
9. Having an affair with a married man/woman. (E)
10. Passing off somebody else's work as your own. (E)
11. Going down a ski run that is beyond your ability. (R)
12. Investing 5% of your annual income in a very speculative stock. (F/I)
13. Going whitewater rafting at high water in the spring. (R)
14. Betting a day's income on the outcome of a sporting event (F/G)
15. Engaging in unprotected sex. (H/S)
16. Revealing a friend's secret to someone else. (E)
17. Driving a car without wearing a seat belt. (H/S)
18. Investing 10% of your annual income in a new business venture. (F/I)
19. Taking a skydiving class. (R)
20. Riding a motorcycle without a helmet. (H/S)
21. Choosing a career that you truly enjoy over a more secure one. (S)
22. Speaking your mind about an unpopular issue in a meeting at work. (S)
23. Sunbathing without sunscreen. (H/S)
24. Bungee jumping off a tall bridge. (R)
25. Piloting a small plane. (R)
26. Walking home alone at night in an unsafe area of town. (H/S)
27. Moving to a city far away from your extended family. (S)
28. Starting a new career in your mid-thirties. (S)
29. Leaving your young children alone at home while running an errand. (E)
30. Not returning a wallet you found that contains $200. (E)

Note. E = Ethical, F = Financial, H/S = Health/Safety, R = Recreational, and S = Social.
