# Supplementary material for: The impact of emotional intelligence and personality traits on the occurrence of unsafe behaviors and needle stick injuries among the nurses
Source: Heliyon. 2022 May 30;8(6):e09584. doi: 10.1016/j.heliyon.2022.e09584 (PMC9344315; doi:10.1016/j.heliyon.2022.e09584)
Supplement: Goleman_emotional_intelligence_test_-_2-10-12 [file mmc4.pdf]

Annex E

The *Practical* EQ Emotional Intelligence Self-Assessment

This self-assessment questionnaire is designed to get you thinking about the various competences of emotional intelligence as they apply to your life. It does not pretend to be a validated psychometric test and the answers you give might vary depending on your mood when you take it.

It is based on the five-competency model of emotional intelligence by Daniel Goleman in the book Emotional Intelligence.

How to complete the questionnaire

Complete each competency page (example below) and use the last page to chart your scores.

1. I can explain my actions:					
Almost Never	Rarely	Sometimes	Usually	Almost Always	
☐	☐	☐	☒	☐	
0	1	2	3	4	**3**
**2. Other people don't see me as I see myself:**					
Almost Never	Rarely	Sometimes	Usually	Almost Always	
☐	☐	☒	☐	☐	
4	3	2	1	0	**2**
**3. I understand the feedback that others give me:**					
Almost Never	Rarely	Sometimes	Usually	Almost Always	
☐	☐	☐	☒	☐	
0	1	2	3	4	**3**
**4. I can describe accurately what I am feeling:**					
Almost Never	Rarely	Sometimes	Usually	Almost Always	
☐	☐	☐	☒	☐	
0	1	2	3	4	**3**
**5. Things that happen in my life make sense to me:**					
Almost Never	Rarely	Sometimes	Usually	Almost Always	
☐	☐	☐	☐	☒	
0	1	2	3	4	**4**
				Total for Self-Awareness:	**15**

©Coaching Leaders Ltd 2012. This product is free for self-assessment only. To use it with others, you must purchase a license from: www.coachingleaders.co.uk/emotional-intelligence-test

Self-Awareness

1. I can explain my actions:					
Almost Never ☐	Rarely ☐	Sometimes ☐	Usually ☐	Almost Always ☐	
0	1	2	3	4	☐
2. Other people don't see me as I see myself:					
Almost Never ☐	Rarely ☐	Sometimes ☐	Usually ☐	Almost Always ☐	
4	3	2	1	0	☐
3. I understood the feedback that others gave me:					
Almost Never ☐	Rarely ☐	Sometimes ☐	Usually ☐	Almost Always ☐	
0	1	2	3	4	☐
4. I can describe accurately what I am feeling:					
Almost Never ☐	Rarely ☐	Sometimes ☐	Usually ☐	Almost Always ☐	
0	1	2	3	4	☐
5. Things that happen in my life make sense to me:					
Almost Never ☐	Rarely ☐	Sometimes ☐	Usually ☐	Almost Always ☐	
0	1	2	3	4	☐
				Total for Self-Awareness:	☐

Emotional Self-Awareness is the ability to recognise what you are feeling, understanding your habitual emotional responses to events and recognising how your emotions affect your behaviour and performance.

When you are self-aware, you see yourself as others see you and have a good sense of your own abilities and current limitations.

©Coaching Leaders Ltd 2012. This product is free for self-assessment only. To use it with others, you must purchase a license from: www.coachingleaders.co.uk/emotional-intelligence-test

Self-Management

1. I can stay calm, even in difficult circumstances:				
Almost Never ☐	Rarely ☐	Sometimes ☐	Usually ☐	Almost Always ☐
0	1	2	3	4
2. I am prone to outbursts of rage:				
Almost Never ☐	Rarely ☐	Sometimes ☐	Usually ☐	Almost Always ☐
4	3	2	1	0
3. I feel miserable:				
Almost Never ☐	Rarely ☐	Sometimes ☐	Usually ☐	Almost Always ☐
4	3	2	1	0
4. I get irritated by things, other people or myself:				
Almost Never ☐	Rarely ☐	Sometimes ☐	Usually ☐	Almost Always ☐
4	3	2	1	0
5. I get carried away and do things I regret:				
Almost Never ☐	Rarely ☐	Sometimes ☐	Usually ☐	Almost Always ☐
4	3	2	1	0
				Total for Self-Awareness:

Emotional Self-Management is the ability to stay focused and think clearly even when experiencing powerful emotions.

Being able to manage your own emotional state is essential for taking responsibility for your actions and can save you from hasty decisions that you later regret.

©Coaching Leaders Ltd 2012. This product is free for self-assessment only. To use it with others, you must purchase a license from: www.coachingleaders.co.uk/emotional-intelligence-test

Motivation

1. I am clear about my goals for the future:					
Almost Never ☐	Rarely ☐	Sometimes ☐	Usually ☐	Almost Always ☐	
0	1	2	3	4	☐
2. My career is moving in the right direction:					
Almost Never ☐	Rarely ☐	Sometimes ☐	Usually ☐	Almost Always ☐	
0	1	2	3	4	☐
3. I find it hard to maintain my enthusiasm when I encounter setbacks:					
Almost Never ☐	Rarely ☐	Sometimes ☐	Usually ☐	Almost Always ☐	
4	3	2	1	0	☐
4. I feel excited when I think of my goals:					
Almost Never ☐	Rarely ☐	Sometimes ☐	Usually ☐	Almost Always ☐	
0	1	2	3	4	☐
5. I act consistently to move towards my goals:					
Almost Never ☐	Rarely ☐	Sometimes ☐	Usually ☐	Almost Always ☐	
0	1	2	3	4	☐
Total for Self-Awareness:					☐

Motivation is the ability to use your deepest emotions to move and guide you towards your goals. This ability enables you to take the initiative and to persevere in the face of obstacles and setbacks.

©Coaching Leaders Ltd 2012. This product is free for self-assessment only. To use it with others, you must purchase a license from: www.coachingleaders.co.uk/emotional-intelligence-test

Empathy

1. My colleagues are uncommunicative:					
Almost Never ☐	Rarely ☐	Sometimes ☐	Usually ☐	Almost Always ☐	
4	3	2	1	0	☐
2. I get on well with each of my work colleagues:					
Almost Never ☐	Rarely ☐	Sometimes ☐	Usually ☐	Almost Always ☐	
0	1	2	3	4	☐
3. I find it easy to "read" other people's emotions:					
Almost Never ☐	Rarely ☐	Sometimes ☐	Usually ☐	Almost Always ☐	
0	1	2	3	4	☐
4. It's unpredictable how my colleagues will feel in any given situation:					
Almost Never ☐	Rarely ☐	Sometimes ☐	Usually ☐	Almost Always ☐	
4	3	2	1	0	☐
5. People choose to work with me in preference to equally-talented colleagues:					
Almost Never ☐	Rarely ☐	Sometimes ☐	Usually ☐	Almost Always ☐	
0	1	2	3	4	☐
				Total for Self-Awareness:	☐

Empathy is the ability to sense, understand and respond to what other people are feeling.

Self-awareness is an essential underpinning of empathy. If you are not aware of your own emotions, you will not be able to read the emotions of others.

©Coaching Leaders Ltd 2012. This product is free for self-assessment only. To use it with others, you must purchase a license from: www.coachingleaders.co.uk/emotional-intelligence-test

Relationship Management

1. I encounter difficult people:				
Almost Never ☐	Rarely ☐	Sometimes ☐	Usually ☐	Almost Always ☐
4	3	2	1	0
2. I am comfortable talking to anyone:				
Almost Never ☐	Rarely ☐	Sometimes ☐	Usually ☐	Almost Always ☐
0	1	2	3	4
3. I achieve win/win outcomes:				
Almost Never ☐	Rarely ☐	Sometimes ☐	Usually ☐	Almost Always ☐
0	1	2	3	4
4. I feel uncomfortable when other people get emotional:				
Almost Never ☐	Rarely ☐	Sometimes ☐	Usually ☐	Almost Always ☐
4	3	2	1	0
5. I get impatient with incompetent people:				
Almost Never ☐	Rarely ☐	Sometimes ☐	Usually ☐	Almost Always ☐
4	3	2	1	0
				Total for Relationship Management: ☐

Relationship Management is the ability to manage, influence and inspire emotions in others.

Being able to handle emotions in relationships and being able to influence and inspire others are essential foundation skills for successful teamwork and leadership.

©Coaching Leaders Ltd 2012. This product is free for self-assessment only. To use it with others, you must purchase a license from: www.coachingleaders.co.uk/emotional-intelligence-test

Total Scores

	Self-Awareness	Self-Management	Motivation	Empathy	Relationship Management
20					
19					
18					
17					
16					
15					
14					
13					
12					
11					
10					
9					
8					
7					
6					
5					
4					
3					
2					
1					
0					
Totals:					

Key

For each area, write the total in the bottom line and shade in the box against the appropriate number to give a graphical representation of your overall score.

14-20	This area is a strength for you
7-13	Some attention given to the aspects of this area you feel are weakest will pay dividends
0-6	This is an area you need to give priority to developing

You can find practical suggestions on how to develop each competency area in the How to Develop Your Emotional Intelligence guide at www.practicaleq.com/products/

©Coaching Leaders Ltd 2012. This product is free for self-assessment only. To use it with others, you must purchase a license from: www.coachingleaders.co.uk/emotional-intelligence-test
