# Supplementary material for: The impact of emotional intelligence and personality traits on the occurrence of unsafe behaviors and needle stick injuries among the nurses
Source: Heliyon. 2022 May 30;8(6):e09584. doi: 10.1016/j.heliyon.2022.e09584 (PMC9344315; doi:10.1016/j.heliyon.2022.e09584)
Supplement: demographic questionnire [file mmc5.pdf]

Annex A

Demographic questionnaire

Name: ………………………………...

Department: ………………………….

Age: …………………………………. (year)

Work experience: …………………….. (year)

Shift number per month: ……………...
