# Supplementary material for: The impact of emotional intelligence and personality traits on the occurrence of unsafe behaviors and needle stick injuries among the nurses
Source: Heliyon. 2022 May 30;8(6):e09584. doi: 10.1016/j.heliyon.2022.e09584 (PMC9344315; doi:10.1016/j.heliyon.2022.e09584)
Supplement: BussPerry_agression_questionnaire_scoring [file mmc6.pdf]

Annex D

Aggression Questionnaire (Buss & Perry, 1992)

Instructions:

Using the 5 point scale shown below, indicate how uncharacteristic or characteristic each of the following statements is in describing you. Place your rating in the box to the right of the statement.

1 = extremely uncharacteristic of me
2 = somewhat uncharacteristic of me
3 = neither uncharacteristic nor characteristic of me
4 = somewhat characteristic of me
5 = extremely characteristic of me

1.	Some of my friends think I am a hothead	☐	A
2.	If I have to resort to violence to protect my rights, I will.	☐	PA
3.	When people are especially nice to me, I wonder what they want.	☐	H
4.	I tell my friends openly when I disagree with them.	☐	VA
5.	I have become so mad that I have broken things.	☐	PA
6.	I can't help getting into arguments when people disagree with me.	☐	VA
7.	I wonder why sometimes I feel so bitter about things.	☐	H
8.	Once in a while, I can't control the urge to strike another person.	☐	PA
9.*	I am an even-tempered person.	☐	A
10.	I am suspicious of overly friendly strangers.	☐	H
11.	I have threatened people I know.	☐	PA
12.	I flare up quickly but get over it quickly.	☐	A
13.	Given enough provocation, I may hit another person.	☐	PA
14.	When people annoy me, I may tell them what I think of them.	☐	VA
15.	I am sometimes eaten up with jealousy.	☐	H
16.*	I can think of no good reason for ever hitting a person.	☐	PA
17.	At times I feel I have gotten a raw deal out of life.	☐	H
18.	I have trouble controlling my temper.	☐	A
19.	When frustrated, I let my irritation show.	☐	A
20.	I sometimes feel that people are laughing at me behind my back.	☐	H
21.	I often find myself disagreeing with people.	☐	VA
22.	If somebody hits me, I hit back.	☐	PA
23.	I sometimes feel like a powder keg ready to explode.	☐	A
24.	Other people always seem to get the breaks.	☐	H
25.	There are people who pushed me so far that we came to blows.	☐	PA

26.	I know that “friends” talk about me behind my back.	☐	H
27.	My friends say that I’m somewhat argumentative.	☐	VA
28.	Sometimes I fly off the handle for no good reason.	☐	A
29.	I get into fights a little more than the average person.	☐	PA

Scoring

The two questions with the asterisk are reverse scored.

The Aggression scale consists of 4 factors, Physical Aggression (PA), Verbal Aggression (VA), Anger (A) and Hostility (H). The total score for Aggression is the sum of the factor scores.

References

Buss, A.H., & Perry, M. (1992). The Aggression Questionnaire. *Journal of Personality and Social Psychology*, *63*, 452-459.
